# Levosimendan’s Effects on Length-Dependent Activation in Murine Fast-Twitch Skeletal Muscle

**DOI:** 10.3390/ijms25116191

**Published:** 2024-06-04

**Authors:** Michael Haug, Mena Michael, Paul Ritter, Larisa Kovbasyuk, Maria Eleni Vazakidou, Oliver Friedrich

**Affiliations:** 1 Institute of Medical Biotechnology, Department of Chemical and Biological Engineering, Friedrich-Alexander-University Erlangen-Nürnberg, Paul-Gordan Str. 3, 91052 Erlangen, Germany; mena.michael@fau.de (M.M.); paul.p.ritter@fau.de (P.R.); larysa.mokhir@fau.de (L.K.); maria.eleni.vazakidou@fau.de (M.E.V.); oliver.friedrich@fau.de (O.F.); 2 Erlangen Graduate School in Advanced Optical Technologies (SAOT), Friedrich-Alexander-University Erlangen-Nürnberg, Paul-Gordan-Str. 6, 91052 Erlangen, Germany; 3 School of Biomedical Sciences, University of New South Wales, Wallace Wurth Building, 18 High St., Sydney, NSW 2052, Australia

**Keywords:** levosimendan, single muscle fibre, calcium sensitivity, stress–strain, *MyoRobot*

## Abstract

Levosimendan’s calcium sensitizing effects in heart muscle cells are well established; yet, its potential impact on skeletal muscle cells has not been evidently determined. Despite controversial results, levosimendan is still expected to interact with skeletal muscle through off-target sites (further than troponin C). Adding to this debate, we investigated levosimendan’s acute impact on fast-twitch skeletal muscle biomechanics in a length-dependent activation study by submersing single muscle fibres in a levosimendan-supplemented solution. We employed our *MyoRobot* technology to investigate the calcium sensitivity of skinned single muscle fibres alongside their stress–strain response in the presence or absence of levosimendan (100 µM). While control data are in agreement with the theory of length-dependent activation, levosimendan appears to shift the onset of the ‘descending limb’ of active force generation to longer sarcomere lengths without notably improving myofibrillar calcium sensitivity. Passive stretches in the presence of levosimendan yielded over twice the amount of enlarged restoration stress and Young’s modulus in comparison to control single fibres. Both effects have not been described before and may point towards potential off-target sites of levosimendan.

## 1. Introduction

Levosimendan is a calcium sensitizing drug that has been used to treat heart failure for over a decade [1,2]. Its primary mechanism of action involves binding to the calcium-bound form of cardiac troponin C (cTnC) and enhances the stability of the cTnC–calcium complex. Thereby, levosimendan increases the affinity of cTnC and sensitizes the troponin complex to calcium ions leading to increased contractility and improved cardiac function [3]. In addition, levosimendan has vasodilative effects that reduce afterload, and it was shown to improve renal function and decrease inflammation [4,5]. In vivo, the parent drug (levosimendan) acts quickly but has a comparatively short half-time (∼1 h) as it is metabolized in the liver [6]. Yet, the long-term effects in vivo are mediated by the long-lasting metabolite OR-1896 (half-time ∼70–80 h) which has the same effect and similar mechanism of action as the parent drug [6,7,8]. While levosimendan is primarily used in the treatment of heart failure, recent studies suggest that the drug may also directly affect skeletal muscle contractility [9,10]. Regarding calcium sensitivity, cardiac muscle is reported to be slightly less calcium sensitive (e.g., murine cardiac myocytes pCa_50_ ∼5.6 [11]) than skeletal muscle (ranging from pCa_50_ ∼5.6 for both slow type-1, and fast type-2 twitch fibres [12] to pCa_50_ ∼6.2 for slow-twitch fibres [13], and pCa_50_ ∼5.9 for fast-twitch fibres [13] from rodents). This indicate a potentially larger amplification by administering the drug to cardiac muscle. Yet, levosimendan was also shown to significantly improve the calcium sensitivity of diaphragm muscle fibres in rats, which suggests that the drug may also affect skeletal muscle cells [14], potentially through off-target effects on proteins involved in calcium regulation other than TnC. For example, levosimendan was shown to interact with the ryanodine receptor [15,16], which is involved in the release of calcium from the sarcoplasmic reticulum (SR) in skeletal muscle, and an interaction of the drug with calmodulin is likewise supposed to explain the sensitizing effects [17]. However, the effects of levosimendan on skeletal muscle are not well understood and may be more complex than initially thought. Therefore, further experimental studies are needed to investigate this potential.

Skeletal muscle is a crucial tissue that enables movement and maintains posture. Muscle weakness presents a common symptom in many diseases, including neuromuscular disorders, heart failure, and ageing [18,19,20]. Levosimendan binds to cTnC, increasing its calcium affinity and thereby enhancing cardiac contractility [3,10,16]. It is possible that levosimendan has a similar effect on skeletal muscle troponin C (sTnC) or related proteins involved in calcium regulation, leading to improved calcium sensitivity and thus, muscle function [2,10]. This could have important implications for disease treatment, e.g., administering levosimendan to improve muscle function in patients with neuromuscular disorders [9,21] or to enhance recovery after muscle injury.

A study on skeletal muscle (diaphragm) suggested that the slow-twitch isoform of TnC is similar to the cardiac isoform, explaining levosimendan’s interaction [14]. Other mechanisms might include off-target interactions with the ryanodine receptor [15,16] or with titin, which influences muscle biomechanics and is calcium-sensitive [22,23,24]. Conclusively, the presently suspected protein targets of levosimendan are all involved in cellular calcium regulation (calcium-dependent force generation and sensitivity) and therefore, are responsive to changes in length-dependent activation (LDA), e.g., a higher calcium sensitivity at longer sarcomere lengths [25,26]. LDA is believed to arise from an optimal sarcomere spacing that facilitates actin–myosin binding and calcium docking to TnC, thus increasing muscular calcium sensitivity. In addition to actin and myosin, titin also seems to play a major role in LDA [27,28,29,30] by modifying muscle fibre stiffness in the A-band in active contraction and passive stretching [24]. As such, the potential involvement of titin in the mechanism of action of levosimendan may provide an additional explanation for the sensitizing effects seen in the diaphragm [14].

To investigate this, we employed our automated *MyoRobot* biomechatronics platform [31,32] to study the acute effects of both LDA and LDA+levosimendan on single muscle fibre active and passive biomechanics. To rule out the possibility that similar isoforms of cTnC and slow-twitch sTnC [21,33] contribute to our results, we investigated single fibres from fast-twitch *M. extensor digtorum longus* (EDL, >90% type II fibres [34]). With our *MyoRobot* technology, we carried out automated recordings of muscle contractility (calcium-saturated induced force), calcium sensitivity (pCa-force curves), and stress–strain relationships at different sarcomere lengths (SLs) in the presence (supplemented at 100 µM in each respective bioactive solution) or absence of levosimendan, thus providing insights into the functional implications of the drug on skeletal muscle. Overall, the potential effects of levosimendan on skeletal muscle are an intriguing area of research that could have important clinical implications.

## 2. Results

### 2.1. Levosimendan Improves Maximum Ca^2+^-Saturated Force Generation at High Sarcomere Lengths, without a Sensitizing Effect

**Figure 1:**
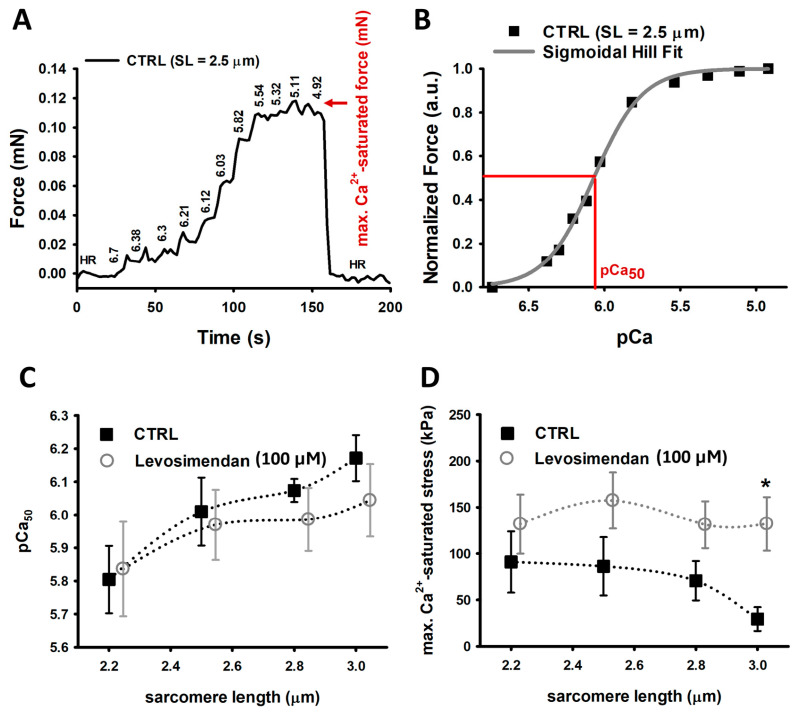
Ca^2+^ sensitivity and maximum Ca^2+^-saturated force recordings. (**A**), Representative force–pCa recording of a CTRL single muscle fibre at 2.5 µm SL. (**B**), by plotting the steady-state force levels against their respective pCa value, a Hill fit’s inflection point represents the pCa_50_ value. Parameters: b (Hill coefficient) = 2.118 and c = 8.385 × 10^−7^ with —log_10_(c) = 6.07 = pCa_50_. (**C**), Ca^2+^ sensitivity recordings yield a gradually increasing pCa_50_ value in agreement with LDA. Both groups display a similar Ca^2+^ sensitivity. (**D**), Maximum Ca^2+^-saturated force is slightly enhanced in single fibres exposed to 100 µM levosimendan vs. control fibres for SLs from 2.2 µm to 2.8 µm. At 3.0 µm SL, single fibres in levosimendan-enriched solution produce significantly more force than untreated fibres. The latter reproduced a force decline in agreement with the descending limb of LDA. Data points were slightly shifted around each other at same SL for visualisation purposes only. The dotted curves connecting the data visualize potential trends but do not explicitly result from any fit procedure. Error bars represent standard error. n: 3–9, valid for both panels and study conditions. * *p* ≤ 0.05.

Our experiments showed that the pCa_50_ value gradually increases with the SL in EDL single muscle fibre (Figure 1C). We observed an increase from ∼5.8 to ∼6.0 in both control single fibres and single fibres exposed to levosimendan-enriched solutions, yet without a significant difference between both groups. Interestingly, when exposing the skinned fibre preparation to a Ca^2+^-rich solution (HA), we detected a significantly improved active stress (force per area) generation for fibres at 3.0 µm SL in the levosimendan-enriched solution and an overall tendency towards higher stress generation (Figure 1D). While control fibres reproduced the descending limb of LDA at 3.0 µm SL, fibres activated in the levosimendan-containing solution maintained a similar tension as for shorter SLs.

### 2.2. Passive Restoration Stress and Young’s Modulus Are Enhanced in Single Fibres Stretched in Levosimendan-Supplemented Solution

**Figure 2:**
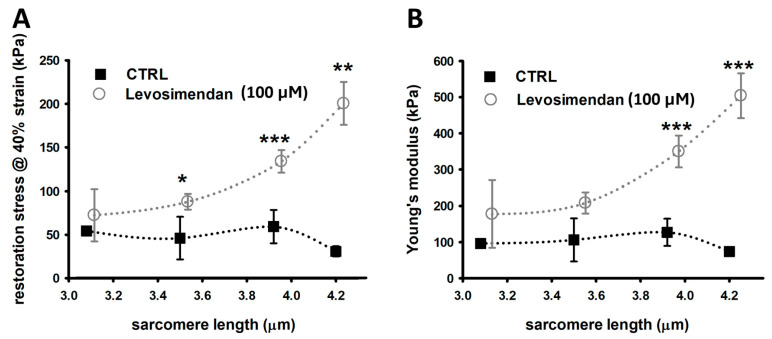
Stress–strain relationship recordings yield an over twice enlarged axial stiffness in single fibres in the presence of levosimendan. (**A**), Maximum passive restoration force was measured at 40% strain. Single EDL fibres exposed to levosimendan solution produce notably more strain over controls, which was significant for larger SLs. (**B**), Similarly, the Young’s moduli of levosimendan-exposed single fibres are much larger. Untreated control fibres are more compliant and display a slow but gradual increase in Young’s modulus with larger SLs, in agreement with passive LDA. This effect is much more pronounced for single fibres in the levosimendan-enriched solution. Data points were slightly shifted at the same SL for visualisation purposes only. The dotted curves connecting the data visualize potential trends but do not explicitly result from any fit procedure. Error bars represent standard error. n: 3–9, valid for both panels and study conditions. * *p* ≤ 0.05. ** *p* ≤ 0.01, *** *p* ≤ 0.005.

Stress–strain relationships were assessed to determine the maximum restoration stress at 140% elongation (40% strain). Maximum restoration stress and the Young’s modulus of untreated fibres slowly but gradually increased with greater SL, according to the passive behaviour of LDA, except for very large SLs of 4.2 µm (Figure 2A,B). This increase was more pronounced in single fibres stretched in a levosimendan-supplemented solution. They seem to display a somewhat exponential increase in maximum restoration stress and Young’s modulus with significantly larger values, particularly at SLs beyond 3.8 µm. As such, single EDL fibres appear to be stiffer in the presence of levosimendan.

## 3. Discussion

In this study, we investigated the effects of LDA [35] at the single-cell level in combination with levosimendan, a common cardiac Ca^2+^ sensitizer. While usually interacting with cTnC, its effects on skeletal muscle fibres are still being debated. Statements in the literature vary, with different authors reporting no effect on the slow-twitch muscle of nemaline myopathy patients [21], an improved Ca^2+^ sensitivity in rat diaphragms [14], enhanced contractility in the human diaphragm [9], an increased grip strength in mice [10], no effects on skeletal muscle at all [21], and that there remains indications that an effect on slow-twitch muscle still exists [2,36]. While in the heart, levosimendan was shown to improve health, continued research suggests potential off-target effects on other calcium-regulating proteins that could also translate to effects in skeletal muscle (e.g., ryanodine 1 receptor [15,16], calmodulin [17]. While other sarcomere proteins like nebulin also contribute to the calcium sensitivity of skeletal muscle [37] and may present potential off-target sites, studies indicate no change in force generation between fibres with intact or mutated nebulin in the presence/absence of levosimendan [21]. Until the present, most levosimendan studies on skeletal muscle focused on active contractility in slow-twitch muscle; therefore, we investigated potential effects on active and passive biomechanics of fast-twitch muscle in an LDA study.

In active contractility, LDA is expressed by, e.g., a higher Ca^2+^ sensitivity at a longer SL [25,38]. The increased sarcomere spacing is thought to facilitate Ca^2+^–TnC docking and actin–myosin interaction. Both untreated control EDL fibres and those incubated in the levosimendan solution display a gradual increase in pCa_50_, with no major difference between both groups. The values ranging from 5.8 to 6.2 compare well with previously published data [20,39,40]. Although in LDA Ca^2+^ sensitivity increases with SL, the amount of generated force follows a bell-shaped relationship, peaking at an optimum SL between 2.3 µm to 2.5 µm in mice [41]. Beyond this SL, maximum contractile force decreases; this is described as the ‘descending limb’ [42,43]. The onset of such decrease is seen in the actively generated stress upon Ca^2+^ exposure in skinned control muscle fibres at 3.0 µm SL. Interestingly, this is not the case for EDL fibres immersed in a levosimendan-supplemented Ca^2+^-rich solution: the fibres display a significant increase in force and appear to shift the descending limb towards higher SLs. While in heart muscle, levosimendan causes an overall improvement of contractile function due to Ca^2+^ sensitization [44], such correlation seems implausible for skeletal single muscle fibres based on our data. To our knowledge, no indication of levosimendan shifting the onset of the descending limb of active contractility has been described in the literature. Yet, to identify whether this is a systematic effect, extended investigations including even larger SLs are needed.

The passive biomechanics properties of single fibres in the presence of levosimendan yield an axial fibre stiffening and enlarged restoration stress at 40% strain. Usually, the response to stress in LDA is largely determined by titin [27,28,29,30] that modifies fibre stiffness, particularly within the A-band related proteins [24]. The restoration force/stress in response to stretch will therefore, consistently increase with SL up to rupture [45]. Myofibrils, single fibres, and whole EDL muscle typically produce between ∼40 to 70 kPa passive restoration stress from 3.0 µm to 4.0 µm [40,46,47,48] which is in agreement with data from our control group; the axial Young’s modulus can easily reveal values that are twice of those ($E_{mod}=\frac{stress}{strain}=\frac{40\mathrm{to}70\;\mathrm{kPa}}{0.4}∼$100–200 kPa). This seems plausible given the fact that the Young’s modulus of soleus single muscle fibres scales between 400–600 kPa [48] and are considered twice as stiff as EDL single muscle fibres [49]. Interestingly, in the presence of levosimendan, EDL single muscle fibres produced over twice the restoration stress and display a significantly higher Young’s modulus. During passive stretch, sarcomeres are thought to somehow ‘sense’ titin stiffness by structural rearrangements or conformational changes of proteins within the actin–myosin overlap zone [30]. Although titin’s intrinsic stiffness also depends on Ca^2+^ levels [23,50], a sensitizing effect of levosimendan on titin’s potential Ca^2+^ binding sites is theoretically possible, but can be ruled out here, since the entire experiment was performed in the absence of Ca^2+^. It remains speculative as to whether the stiffening originates from a direct interaction with sTnC and an associated stiffening of its interaction with actin; yet, it raises the important question of whether levosimendan would potentiate the stiffening effects of Ca^2+^ ions on titin [51], which were shown to increase titin’s rupture forces within the N2A region and Ig domains [23]. This will require the execution of active stretches in the presence of levosimendan and will present an interesting future research question. So far, considering the absence of Ca^2+^ ions in our stress–strain relationship experiments, we currently cannot explain the observed stiffness increase, and more detailed experiments may provide an answer.

**Figure 3:**
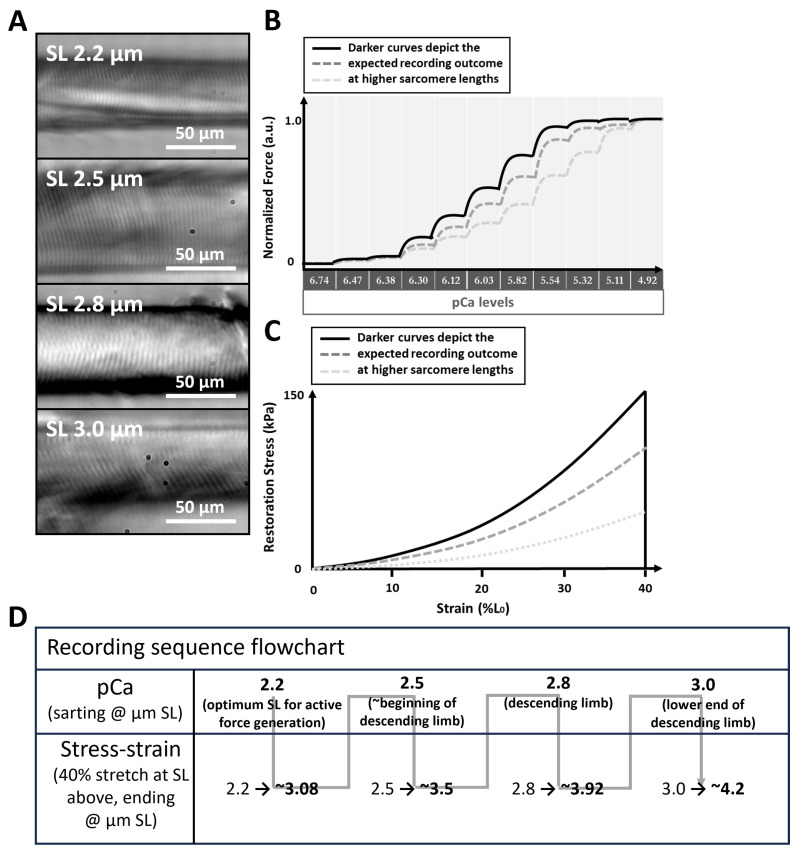
LDA study design. (**A**), single muscle fibres were analysed at distinct SLs starting at 2.2 µm and iteratively increasing SL up to 3.0 µm. The images were obtained with the *MyoRobot’s* integrated optics system. The experimental procedures shown in (**B**) (force–pCa recording) and (**C**) (stress–strain relationship) were carried out in the presence or absence of 100 µM levosimendan. In force–pCa recordings, an increasing SL leads to the sensitization of the myofibrillar contractile apparatus. In stress–strain curves, an increasing SL results in a steeper increase in restoration stress. (**D**), flow chart of the recording sequence.

It must be noted that there are limitations to this study which must be addressed in future investigations. First, levosimendan (as in this study) is commonly diluted in a DMSO medium, which was suggested to depress muscle contractility [52]. As such, it is plausible that the effects of 100 µM levosimendan on maximum Ca^2+^-saturated stress might be underestimated due to potential remains of the DMSO vehicle, and a dilution in, e.g., glucose solution should be employed as cross-reference. In addition, 100 µM levosimendan represents the top end of externally applied concentrations to skeletal muscle in the literature [14]. In patients with heart failure, levosimendan is usually administered in doses up to 0.2 µg/kg/min, translating to roughly 0.4 nM [53] in the blood serum. Whether concentrations in the µM range are factually present in patient’s muscle tissue remains to be investigated and must be related to experiments where levosimendan is diluted, e.g., in glucose solution to avoid an underestimation of its effects and thus define a dosage at which effects can be expected in the body. Lastly, at the time of initiating this study, we expected the parameter ‘sarcomere length’ to play the predominant role in our LDA assessment, for which we decided on an experiment layout as in Figure 3D. With this design, we were able to conduct paired statistics considering the parameter ‘sarcomere length’. With knowledge of the here-presented data, a follow up study should include investigating the parameter ‘levosimendan presence/absence/concentration’ at a single predefined SL to elucidate the effects exclusive to levosimendan.

## 4. Materials and Methods

This study was conducted in accordance with the approved animal experimentation guidelines at the Friedrich-Alexander-University Erlangen-Nürnberg (TS06/2016). The German regulations for the care of laboratory animals and the guidelines of the Federation of European Laboratory Animal Sciences Associations apply.

### 4.1. Animal Handling and Muscle Fibre Preparation

All animals for this study were obtained from a tissue sharing collaboration with local institutes and were male C57BL/6 mice of approx. 30 g body weight and aged between 15–21 weeks. Five mice were assigned to each study cohort (CTRL group or in vitro exposition to levosimendan). After sedating the animal with 5% isoflurane and performing cervical dislocation, the hind limbs were cut off. Samples were transferred into Ringer’s solution (see Section 4.3 Bioactive Solutions.), and the *M. extensor digitorum longus* (EDL, >90% type II fibres [34]) was manually isolated (scissors and tweezers (Dumont #5): FST, Foster City, CA, USA) under a stereomicroscope (Olympus SZ-X7, Olympus, Shinjuku-ku, Japan). The muscle was pinned onto a Polydimethylsiloxane (PDMS, Sylgard, Dow Corning, Midland, MI, USA)-coated Petri dish under slight pre-stretch. Ringer’s solution was replaced by a high-potassium solution (HKS, see Section 4.3 ‘Bioactive solutions’), and the preparation was allowed to rest and equilibrate for 30 min. Whenever the preparation was allowed to rest, it was bubbled with atmospheric air. HKS permanently depolarizes the membrane and inactivates sodium channels after an initial contraction. Single muscle fibre segments were isolated and tied to two silk micro knots. The fibre was transferred in HKS to the *MyoRobot* and mounted between the pins of a force transducer (FT) and a voice coil (VC) actuator (a linear actuator working with the principle of Lorentz force) by tightening the knots and lowering it in the *idle well* underneath (the idle well contains a 0.01 *v*/*v* ratio of HR in LR solution to buffer Ca^2+^ ions and keep the preparation relaxed, see Section 4.3 Bioactive Solutions). Prior to any physiological recording, the sarcolemma was permeabilized by exposure to a 0.01% (*w*/*v*) saponin solution (20 s) for diffusional access to the myoplasm.

### 4.2. Study Design

To study LDA, the sarcomere length (SL) must be precisely controlled; for this purpose, the *MyoRobot’s* integrated optics system was used. Initially, SL was adjusted to 2.2 µm (considered as the optimum SL overlap in active LDA [54]) by driving the VC at 1 µm/s to elongate the fibre (Figure 3A). Once reached, the fibre was subjected to a force–pCa recording (active biomechanics, Figure 3B) and a stress–strain relationship recording (passive biomechanics, Figure 3C). Subsequently, the same single fibre was stretched to 2.5 µm SL (considered as the beginning of the descending limb [41]), and the procedure repeated until an SL of 3.0 µm (considered as end of the descending limb of active LDA [41]) was reached. In case of fibre rupture, which may occur when the fibre is stretched beyond 4.0 µm SL due to titin disintegration [55], the fibre was excluded from analysis and the process was re-initiated at 2.0 µm SL with a new muscle fibre. A single experiment day either investigated muscle fibre exposed to bioactive solutions *without* or *with* levosimendan, meaning that experiments *with* or *without* levosimendan took place at different days with different single muscle fibres (unpaired data). The recording sequence carried out on each respective day is displayed in Figure 3D. This design allowed us to conduct a complete pCa–force recording followed by a stress–strain recording on the same single fibre covering four distinct sarcomere lengths before moving to the next single fibre. This allowed for self-control experiments that consider the parameter ‘sarcomere length’.

### 4.3. Bioactive Solutions

Solutions for fibre manipulation (calcium activation or relaxation) were used and composed as described in [39]. Their summarized physiological purposes are the following:

- **Ringer’s solution** [56]: isotonic electrolyte solution for tissue dissection, storage, and transfer (before single-fibre isolation);
- **High-potassium solution (HKS)**: permanently depolarises the membrane potential and keeps the muscle preparation in a relaxed, inexcitable state;
- **High-activating solution (HA)**: EGTA-buffered Ca^2+^-rich solution to evoke skinned single muscle fibre maximum contraction at pCa = 4.92;
- **High-relaxing solution (HR)**: calcium-free, EGTA-buffered solution to relax the permeabilized muscle fibre (pCa = 9);
- **Low-relaxing solution (LR)**: calcium-free, HDTA-buffered solution applied after HR exposure to exchange the strong EGTA calcium buffer by the mild buffering agent HDTA.

For calcium sensitivity recordings, HA and HR solutions were mixed to achieve defined total and free calcium concentrations. Mixing ratios were calculated in MAXCHELATOR (developed by Chriss Paton, somapp.ucdmc.ucdavis.edu/pharmacology/bers/maxchelator/) to achieve the distinct pCa steps shown in Figure 3. All solutions were supplemented with 10 µL of creatine kinase to a total of 1 mL in the rack’s wells. On experiment days investigating the potential effects of levosimendan, the drug was added to each well to a total concentration of 100 µM.

### 4.4. Recording Procedure and Data Analysis

All recordings were performed with our robotized *MyoRobot* biomechatronics system [32] at 23 °C room temperature with no external oxygenation in the *MyoRobot*’s wells. Data were sampled at 100 Hz while two linear stages sequentially immersed the single muscle fibre in the bioactive solutions of the rack. A VC was used to stretch the preparation to 140% of its resting length (typically: L_0_ ∼2 mm) at 1 µm/s. Data were stored in a LabVIEW (Laboratory Virtual Instrument Engineering Workbench, National Instruments, TX, USA) internal data format (TDMS) and text format for automated processing using scripts written in RStudio (RStudio Inc., rstudio.com, Boston, MA, USA [57], RRID:SCR_000432) as described in [31].

To assess the calcium sensitivity of the contractile apparatus in the absence of levosimendan (CTRL study group), a single muscle fibre was sequentially exposed to solutions with increasing calcium concentration (decreasing pCa, pCa = —log_10_[Ca^2+^]). The same recording was also carried out on another preparation in the same solutions but supplemented with 100 µM levosimendan (in vitro levosimendan exposed study group). The exact values are displayed in Figure 3B. For each pCa level, the plateau force was analysed, while the steady-state force at pCa = 4.92 was considered as maximum calcium-saturated force (Figure 1A). Normalizing this to the fibre’s cross-sectional area yielded the maximum calcium-saturated stress. To compute the pCa_50_ value, a marker for calcium sensitivity, extracted plateau forces were normalized to maximum calcium-induced force. A Hill fit according to $y=\frac{10^{-bx}}{c^{b}+10^{-bx}}$, with x being pCa, and y being force [58], was fitted to the data (Figure 1B). The optimized c value reflects the Ca_50_ value, and the —log_10_(c) equals the pCa_50_ value.

A stress–strain recording consisted of stretching the single muscle fibre to 140% of its resting length under relaxing and calcium-free conditions. Those experiments were also independently carried out in levosimendan-free (CTRL study group) and levosimendan supplemented solutions (in vitro levosimendan-exposed study group). Commencing at a median SL of 2.2 µm, a stretch by 40% resulted in a median SL of 3.08 at 140% L_0_. These values are associated with a margin of error (roughly ±0.1 µm) arising from a Gaussian distribution of all SLs within a field-of-view around the median SL. Such Gaussian distributions of SLs are thoroughly reported in the literature [41,59,60] and extensively discussed in a previous publication [61]. Once 140% resting length was reached, maximum passive axial restoration force was analysed and normalized to the single-fibre cross-sectional area to yield restoration stress. A linear fit applied to the resulting stress–strain curve allowed the extraction of the fibre’s passive axial elastic modulus (Young’s modulus: $E=\frac{\sigma (\epsilon )}{\epsilon }$, with σ being the tensile stress at a specific elongation, and ϵ being the tensile strain).

Data were plotted using Sigma Plot (Systat Software Inc., www.systat.de/SigmaPlot_Produktseite, accessed on 10 April 2024, San Jose, CA, USA, RRID:SCR_003210). All data were analysed for normality by performing a Shapiro–Wilk test. If a normal distribution was not given, a rank-based ANOVA test was conducted and followed by a post-hoc analysis according to Dunn’s median comparison. If data were normally distributed, a one-way ANOVA test following post-hoc analysis according to Bonferroni’s method was applied. If statistical tests confirmed any significant difference, this was indicated by vertical bars in all plots, including an asterisk to indicate the magnitude as follows: * *p* ≤ 0.05, ** *p* ≤ 0.01, *** *p* ≤ 0.005. An absence of such indicators is equivalent to ‘no significant difference’.

## 5. Conclusions

Our investigation delved into the intricate interplay between LDA and levosimendan in the context of skeletal muscle biomechanics. Through a comprehensive investigation, we confirmed that levosimendan’s effects extend beyond its known impact on cardiac muscle. Our findings demonstrate that acute levosimendan exposition induces notable shifts in the descending limb of active contractility, a phenomenon previously unreported in skeletal muscle. Furthermore, we revealed a significant enhancement in axial fibre stiffness and restoration stress under passive stretch conditions in the presence of levosimendan, opening new avenues for understanding the roles of titin and sarcomere mechanics. The identification of levosimendan’s differential effects on cardiac and skeletal muscle underscores the intricate regulatory mechanisms governing contractility. This knowledge may have significant implications for therapeutic strategies targeting muscle-related disorders and conditions. The pronounced modifications in passive biomechanics suggest a potential role of levosimendan in influencing tissue stability and mechanical properties, prompting further investigation into its mechanotransduction pathways and implications for muscle health. However, it must be mentioned that this study involved acute exposure to levosimendan. As such, we cannot conclude on the potential effects of in vivo metabolism of the drug and its potential influence on regulating muscle homeostasis and morphology.

## Data Availability

The data that support the findings of this study are available from the corresponding author, M.H.

## Abbreviations

The following abbreviations are used in this manuscript:

cTnC	Cardiac troponin C
EGTA	Ethylene glycol-bis(β-aminoethyl ether)-N,N,N’,N’-tetraacetic acid
EDL	*M. extensor digitorum longus*
FT	Force transducer
HA	High-activating solution
HDTA	Hexamethylene-diamine tetraacetate
HKS	High-potassium solution
HR	High-relaxing solution
L_0_	Resting length
LDA	Length dependent activation
LR	Low-relaxing solution
PDMS	Polydimethylsiloxane
RyR1	Ryanodine 1 Receptor
SR	Sarcoplasmic reticulum
sTnC	Skeletal muscle troponin C
SL	Sarcomere length
VC	Voice coil
